# Prevalence of MDR pathogens of bacterial meningitis in Egypt and new synergistic antibiotic combinations

**DOI:** 10.1371/journal.pone.0171349

**Published:** 2017-02-16

**Authors:** Mona M. Abdelkader, Khaled M. Aboshanab, Marwa A. El-Ashry, Mohammad M. Aboulwafa

**Affiliations:** 1 Department of Microbiology & Immunology, Faculty of Pharmacy, Ain Shams University, Cairo, Egypt; 2 Department of Clinical and Chemical Pathology, Faculty of Medicine, Ain Shams University, Cairo, Egypt; University of Malaya, MALAYSIA

## Abstract

The aim of this study was identifying bacterial pathogens involved in meningitis, studying their antibiotic resistance profiles, investigating the antibiotic resistance genes as well as evaluating the use of various antibiotic combinations. Antibiotic susceptibility tests were evaluated according to CLSI guidelines. Antibiotic combinations were evaluated by calculating the Fractional Inhibitory Concentration (FIC) index. A total of 71 bacterial isolates were recovered from 68 culture positive CSF specimens. Sixty five of these isolates (91.5%) were recovered from single infection specimens, while 6 isolates (8.4%) were recovered from mixed infection specimens. Out of the 71 recovered isolates, 48 (67.6%) were Gram-positive, and 23 (32.4%) were Gram-negative. Thirty one of the Gram positive isolates were *S*. *pneumoniae* (64.6%, n = 48). Out of the recovered 71 isolates; 26 (36.6%) were multidrug-resistant (MDR) isolates of which, 18 (69.2%) were Gram-negative and 8 (30.8%) were Gram-positive. All MDR isolates (100%) showed resistance to penicillin and ampicillin, however, they showed lower resistance to meropenem (50%), levofloxacin (50%), amikacin (48%), pipercillin-tazobactam (45.8%). Most common antibiotic resistance genes were investigated including: *tem* (21.1%), *shv* (15.8%), *ctx*-*m* (15.8%) coding for TEM-, SHV, CTX-M extended-spectrum beta-lactamases (ESBLs), respectively; *aac(6')-I b*(26.3%) coding for aminoglycoside 6’-N-acetyltransferase type Ib ciprofloxacin resistant variant; and *qnr*A (5.3%) gene coding for quinolone resistance. The DNA sequences of the respective resistance genes of some selected isolates were PCR amplified, analyzed and submitted to the GenBank database under the accession numbers, KX214665, KX214664, KX214663, KX214662, respectively. The FIC values for ampicillin/sulbactam plus cefepime showed either additive or synergistic effect against ten tested Gram-negative MDR isolates, while doxycycline plus levofloxacin combination revealed synergism against two MDR Gram-positive isolates. The results indicate high prevalence of antibiotic resistance among MDR isolates. Therefore, new guidelines should be implemented in Egypt to rationalize the use and avoid the misuse and abuse of antimicrobial agents.

## Introduction

Bacterial meningitis is a life threatening disease that is associated with significant mortality and morbidity [1]. Historically, three major pathogens account for most cases of bacterial meningitis, which includes: *Neisseria (N*.*) meningitidis*, *Streptococcus (S*.*) pneumoniae*, and *Haemophilus (H*.*) influenzae*, they accounted for about 75–80% of cases globally, while the majority of other cases accounted by *Escherichia (E*.*) coli*, *Listeria (L*.*) monocytogenes*, and *Staphylococcus (S*.*) aureus* [2], however bacterial pathogens causing meningitis are evolving constantly, as evidenced by the change in relative occurrence of pneumococcal meningitis in sub-Saharan [3]. The mortality from these bacterial varies from 3 to 21%, according to type of organism [4]. The incidence and case-fatality rates of bacterial meningitis vary according to country, region, pathogen, and age group. Without any treatment, the case-fatality rate can reach 70%, and one in five survivors of bacterial meningitis may be left with permanent disability including hearing loss, neurologic disability, or limb loss [5]. Routine vaccination against three most common causative bacterial pathogens had a considerable effect on the prevalence of bacterial meningitis. However, an estimated 1–2 million cases of bacterial meningitis occur worldwide every year, resulting in 180 000 deaths in children age from one to 59 months in 2010 [6,7]. The epidemiology of acute bacterial meningitis has changed markedly since the introduction of conjugate vaccines [4,8,9]. However, the disease continues to cause a heavy burden even in developed countries, causing substantial morbidity and mortality [1,8]. Early administration of antibiotics save lives, but the globally emerging multidrug resistant bacteria limits the effectiveness of many inexpensive and widely available antimicrobial drugs [10]. The molecular mechanisms of antibiotics resistance have been studied extensively and involved studying genetics and biochemical aspects of bacterial cell function [10–13]. However, most of these mechanisms can be disseminated by one or more distinctive gene transfer mechanisms [14]. Under selective pressure of certain antibiotics, bacterial variants evolve various mechanisms to survive in the presence of these antimicrobial agents. Drug resistant bacteria are usually multi-drug resistant against various structurally different drugs [15]. Although antimicrobial resistance is classically attributed to chromosomal mutations, resistance is mainly associated with extra-chromosomal elements acquired from other bacteria in the environment, such as plasmids. Multidrug-resistant bacteria will continue to persist and spread worldwide, causing clinical failure in treatment of infectious diseases and public health crises [11]. Therefore, in this study we aim to identify the most common bacterial pathogens together with their resistance profile against major antibiotics used in the empirical treatment of bacterial meningitis. In addition, we aimed to investigate the most common genes involved in bacterial resistance and to evaluate use of various antibiotic combinations for possible synergistic activities against the most clinically relevant pathogens causing bacterial meningitis particularly MDR.

## Materials and methods

### Sample collection

A total of 1337 cerebrospinal fluid (CSF) specimens from suspected cases of meningitis were collected over a2-year period from September 2013 to September 2015. From the culture positive CSF specimens, a total of 71 bacterial isolates were recovered from 3 different hospitals; (AbbassiaFeverHospital,54 isolates; Ain Shams University Hospital, 15 isolates and Ain Shams Specialized Hospital, 2 isolates). Microscopical examination of Gram-stained smears was performed. Characteristics of growth on mannitol salt agar, and results of coagulase and catalase tests, were used to identify the Gram-positive isolates as *Staphylococcus aureus* and *Staphylococcus (S*.*) coagulase negative*. Screening for methicillin resistance was done by agar disc diffusion method according to the CLSI using cefoxitin discs (FOX, 30μg). *S*. *pneumoniae* and *Enterococcus* were cultured on blood and chocolate agar. Optochin (OP) test and bile solubility test were performed to identify *S*. *pneumonia*e isolates, while *Enterococci* were identified according to growth characteristics on Bile Esculin Agar (BEA) and upon addition of Pyrrolidonyl Arylamidase (PYR) reagent. Gram negative isolates were cultured mainly on MacConkey’s agar. Several biochemical tests were performed including, triple sugar iron (TSI) test, oxidase test, citrate utilization, urease test, motility indole ornithine (MIO) test, lysine iron agar (LIA) and slide agglutination test, to identify different bacterial species. Identification of *Enterobacteriaceae* and *Pseudomonas* isolates were confirmed using API® 20E identification kit (BioMérieux, France).

### Antimicrobial susceptibility testing and identification of multidrug-resistant (MDR) isolates

The Kirby-Bauer disk diffusion method was used to determine the susceptibility of the recovered clinical isolates to antimicrobial agents and it was carried out as recommended by the Clinical and Laboratory Standards Institute (CLSI) [16]. Isolates that were resistant to three or more classes of antimicrobials were considered as MDR isolates [17] and were selected for further study.

### Determination of minimum inhibitory concentrations of some selected antimicrobial agents for multi-drug resistant isolates

The minimum inhibitory concentrations (MICs) of amikacin, ceftriaxone, cefepime, levofloxacin, gentamicin, ampicillin/sulbactam, ceftazidime, and imipenem were determined for MDR Gram-negative isolates, while for Gram-positive isolates gentamicin and ceftazidime were replaced by vancomycin and doxycycline. This was done by the micro-broth dilution technique described in the CLSI guidelines [16] and in triplicate where average MIC was calculated.

### Antibiotic combinations

The value of the fractional inhibitory concentration (FIC) index as a predictor of synergy has been investigated according to the protocol described by Hsieh,et al. [18].

### DNA extraction from MDR isolates

GeneJet plasmid miniprep kit (ThermoFisher Scientific, USA) was used to extract plasmid DNA from MDR isolates, however no bands were recovered. While chromosomal DNA was extracted from the tested clinical bacterial isolates using PrepMan Ultra Kit (ThermoFisher Scientific, USA) each according to its manufacturer specifications. The extracted DNA was analyzed using agarose gel electrophoresis [19].

### Amplification of some resistance genes by PCR

Amplification of the selected antibiotic resistance genes were carried out via PCR using appropriate primers (Table 1) and either extracted plasmid or chromosomal DNA of tested MDR bacterial isolates as templates. Primers were synthesized by Invitrogen®, UK and supplied by Analysis Co., Egypt. Detection of the amplified products was done by agarose gel electrophoresis and the expected size of DNA fragment was determined as compared to DNA ladder (GeneRuler100bp, ThermoFisher Scientific, USA).

**Table 1:** Target genes and primers sequences, along with expected PCR product size.

gene	Primer sequence (5'-3')	Expected product size (bp)	T_a_ (°C)	Reference
*ctx-m*	P_f_−CGCTTTGCGATGTGCAG	550	47	[20]
	P_r_−ACCGCGATATCGTTGGT			
*shv*	P_f_—GGTTATGCGTTATATTCGCC	867	47	[21]
	P_r_−TTAGCGTTGCCAGTGCTC			
*tem*	P_f_—ATGAGTATTCAACATTTCCG	867	47	[21]
	P_r_−CTGACAGTTACCAATGCTTA			
*aac(6')-Ib or aac(6')-Ib-cr*	P_f_—TTGCGATGCTCTATGAGTGG	458	46	[22]
	P_r_–CGTTTGGATCTTGGTGACCT			
*qnr*	P_f_−GGAAGCCGTATGGATATTATTG	660	51	[23]
	P_r_−CTAATCCGGCAGCACTATTAC			

*ctx-m*, *shv*, *tem*, genes coding for extended-spectrum beta-lactamases (ESBLs); *aac(6')-Ib* gene coding for aminoglycoside 6’-N-acetyltransferase type Ib; *aac(6')-Ib-cr* gene coding for aminoglycoside 6’-N-acetyltransferase type Ib ciprofloxacin resistant variant; *qnr*A, gene coding for quinolone resistance, T_a_, calculated annealing temperatures.

### Sequencing of some selected PCR products

The PCR products were purified using GeneJET^TM^ purification kit at Sigma Scientific Services Company, Egypt. PCR products were sent for sequencing at GATC, Germany using ABI3730 xl DNA sequencer. The obtained sequence files were assembled into the final consensus sequence using StadenPackage program version3 (http://staden.sourceforge.net/) [24].

## Results

### Data analysis

**Fig 1:**
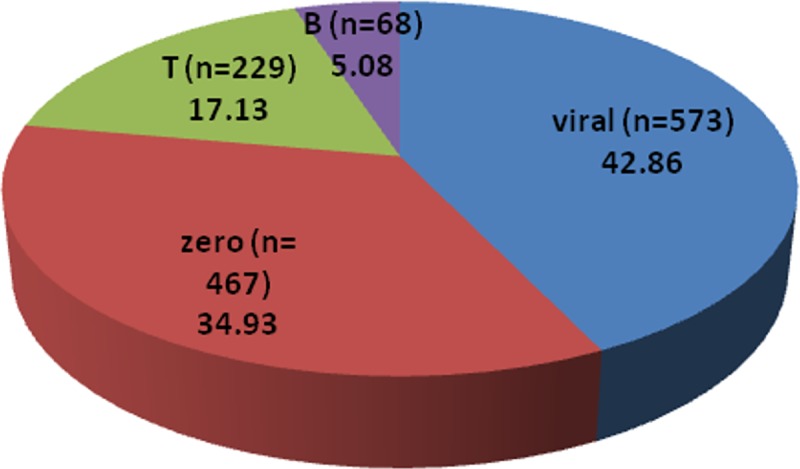
Relative percentage of various CSF specimens collected during the study period (n = 1337). Out of the 68 culture positive specimens, 71 bacterial isolates were recovered from cerebrospinal fluid (CSF); 65 isolates (91.5%) were from specimens with single bacterial species, 6 isolates (8.4%) were from mixed culture. Specimens collected from males were 44(64.7%), while only 24 specimens were collected from females (35.3%). Regarding age, 5 specimens (7.35%) were from infants (age from 1–12 Months), 10 specimens (14.7%) were from children (>1–16 Years) and the rest of the specimens (77.9%) were from adults (>16Years). Using Gram-stain, 48 isolates (67.6%) were found to be Gram-positive and 23 isolates (32.4%) were found to be Gram-negative. The prevalence of different clinical bacterial isolates cultured from the 68 CSF specimens is delineated in Fig 2.

This study was conducted over a period of 2 years from September 2013 to September 2015 where a total of 1,337 CSF specimens were collected (according to Hospitals Official Daily Records). Depending on PMNLs/Lymphocytes ratios(obtained from cell count of conducted microscopically using Hemocytometer; (Assistent, Germany),WBC count (normal level 0–5 cell\mm^3^), protein level (measured by BCA protein assay with normal protein level: 15- 45mg\dl)and glucose levels (measured by Trinder method where the reference values for glucose level 45-75mg\dl), the specimens have been divided in to 4 categories: a) viral (lymph cells exceed PMNLs, protein>45mg\dl, glucose level: normal); b) zero (no cells found); c) turbid no growth (PMNLs exceeds lymphocytes and negative culture, glucose<40mg\dl, protein>50mg\dl); and d) bacterial (PMNLs exceed lymphocytes, glucose <40mg\dl, protein level >50mg\dl and positive culture growth). It has been found that the majority of meningitis cases over the two years period (n = 1337), were possibly viral meningitis (573 cases; 42.86%), followed by cases that have been probably misdiagnosed or have diseases that showed symptomatic similarities with meningitis in which cell count was reported “Zero” (467 cases; 34.93%), followed by cases that showed CSF findings suggesting bacterial meningitis yet showed no positive culture growth represented by letter “T” (turbid no growth; 229 cases; 17.13%), and finally, bacterial meningitis cases that showed positive culture growth represented by letter “B”(68; 5.08%) as shown in Fig 1.

**Fig 2:**
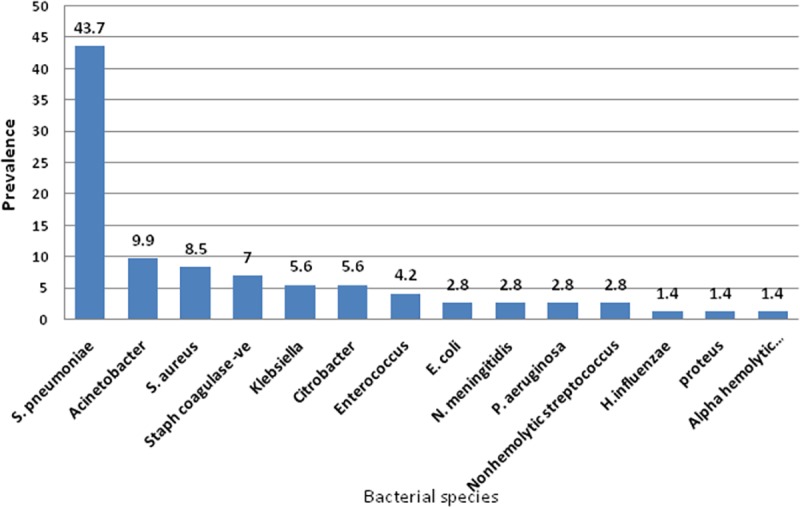
Prevalence of different clinical bacterial isolates cultured from the 68 CSF specimens. Prevalence was expressed as percentage from total count (n = 71).

### Drug susceptibility test

The antimicrobial susceptibility patterns of the bacterial pathogens recovered from the collected CSF specimens of suspected cases of bacterial meningitis that showed positive cultural growth are shown in Table 2. Moreover, the antibiotic susceptibility patterns of the recovered Gram positive and Gram negative isolates are shown in Tables 3 and 4, respectively.

**Table 2:** Antimicrobial susceptibility patterns of meningitis pathogens.

Antimicrobial Agent	Resistance profile												Total Tested isolates
	Sensitive				Intermediate				Resistant				
	Number of:			≈%^(b)^)	Number of:			≈%^(b)^)	Number of:			≈%^(b)^)	
	G+^(a)^	G-^(a)^	total		G+^(a)^	G-^(a)^	total		G+^(a)^	G-^(a)^	total		
**Ampicillin/sulbactam**	31	4	35	64.8	1	2	3	5.6	3	13	16	29.6	54
**Piperacillin/tazobactam**	30	11	41	78.8	0	0	0	0.0	3	8	11	21.2	52
**Ciprofloxacin**	6	12	18	51.4	1	1	2	5.7	6	9	15	42.8	35
**Chloramphenicol**	38	7	45	68.2	1	0	1	1.5	5	15	20	30.3	66
**Penicillin-G**	26	0	26	38.8	0	0	0	0.0	21	20	41	61.2	67
**Cefotaxime**	30	6	36	65.5	0	0	0	0.0	5	14	19	34.5	55
**Vancomycin**	43	nd	43	89.5	0	nd	0	0.0	5	nd	5	10.4	48
**Ceftriaxone**	31	5	36	61.0	0	1	1	1.7	7	15	22	37.3	59
**Amoxicillin/clavulanate**	28	4	32	64.0	0	0	0	0.0	5	13	18	36.0	50
**Amikacin**	9	10	19	59.4	0	1	1	3.1	2	10	12	37.5	32
**Levofloxacin**	35	10	45	70.3	0	2	2	3.1	10	7	17	26.6	64
**Ampicillin**	28	2	30	48.4	0	0	0	0.0	13	19	32	51.6	62
**Gentamicin**	5	11	16	51.6	0	2	2	6.4	6	7	13	41.9	31
**Aztreonam**	0	2	2	33.3	0	0	0	0.0	0	4	4	66.7	6
**Meropenem**	32	13	45	78.9	0	0	0	0.0	3	9	12	21.1	57

nd, not determined; G+, Gram-positive isolates, G-, Gram-negative isolates, **≈%**^**(b)**^, percentage from total number.

**Table 3:** Antimicrobial susceptibility patterns of Gram-positive pathogens.

**S. aureus (N = 6)**			
**Antimicrobial agent**	**Resistant (%)**	**Intermediate (%)**	**Sensitive (%)**
**Penicillin G**	100	0	0
**Ampicillin**	100	0	0
**Ceftriaxone**	50	0	50
**Cefoxitin**	0	0	100
**Levofloxacin**	16.7	0	83.3
**Ciprofloxacin (*n* = 5)**	40	0	60
**Chloramphenicol**	33.3	0	66.7
**Piperacillin / tazobactam**	0	0	100
**Ampicillin / sulbactam**	16.7	16.7	66.7
**Gentamicin**	50	0	50
**Amikacin**	0	0	100
**Meropenem**	0	0	100
**Vancomycin**	16.7	0	83.3
**Coagulase-negative *staphylococci* (N = 5)**			
**Antimicrobial agent**	**Resistant (%)**	**Intermediate (%)**	**Sensitive (%)**
**Penicillin G**	100	0	0
**Ampicillin**	100	0	0
**Cefotaxime**	60	0	40
**Ceftriaxone**	60	0	40
**Levofloxacin**	100	0	0
**Ciprofloxacin**	60	0	40
**Chloramphenicol**	60	0	40
**Piperacillin / tazobactam**	60	0	40
**Ampicillin / sulbactam**	40	0	60
**Amoxicillin / clavulanate**	60	0	40
**Amikacin**	40	0	60
**Gentamicin**	60	0	40
**Meropenem**	60	0	40
**Vancomycin**	40	0	60
***Enterococcus* sp. (N = 3)**			
**Antimicrobial agent**	**Resistant (%)**	**Intermediate (%)**	**Sensitive (%)**
**Penicillin G**	66.7	0	33.3
**Ampicillin**	33.3	0	66.7
**Levofloxacin**	33.3	0	66.7
**Chloramphenicol**	0	33.3	66.7
**Ciprofloxacin**	33.3	33.3	33.3
**Vancomycin**	33.3	0	66.7
**Other Streptococci (N = 3)**			
**Antimicrobial agent**	**Resistant (%)**	**Intermediate (%)**	**Sensitive (%)**
**Penicillin G (*n* = 2)**	50	0	50
**Ampicillin**	33.3	0	66.7
**Cefotaxime (*n* = 2)**	50	0	50
**Chloramphenicol**	0	0	100
**Ceftriaxone**	33.3	0	66.7
**Vancomycin**	33.3	0	66.7

N, total number of isolates, *n*, number of tested isolates, R, resistant, I, intermediate, S, sensitive, %, percentage

**Table 4:** Antimicrobial susceptibility patterns of Gram-negative isolates.

***Enterobacteriaceae* (N = 11)**			
**Antimicrobial agent**	**Resistant (%)**	**Intermediate (%)**	**Sensitive (%)**
**Penicillin G (*n* = 10)**	100	0	0
**Ampicillin**	90.9	0	9.1
**cefotaxime**	54.5	0	45.5
**Ceftriaxone**	63.6	0	36.4
**Levofloxacin**	27.3	18.2	54.5
**Ciprofloxacin (*n* = 10)**	20	10	70
**Chloramphenicol**	54.5	0	45.5
**Piperacillin / tazobactam**	27.3	0	72.7
**Ampicillin / sulbactam**	54.5	9.1	36.4
**Amoxicillin / clavulanate**	63.6	0	36.4
**Gentamicin**	18.2	18.2	63.6
**Amikacin**	36.4	0	63.6
**Meropenem**	27.3	0	72.7
***Acinetobacter spp* (N = 7)**			
**Antimicrobial agent**	**Resistant (%)**	**Intermediate (%)**	**Sensitive (%)**
**Penicillin G**	100	0	0
**Ampicillin**	100	0	0
**cefotaxime**	85.7	0	14.3
**Ceftriaxone**	85.7	14.3	0
**Levofloxacin (*n* = 6)**	66.7	0	33.3
**Ciprofloxacin**	85.7	0	14.3
**Chloramphenicol**	100	0	0
**Piperacillin / tazobactam**	71.4	0	28.6
**Ampicillin / sulbactam**	100	0	0
**Amoxicillin / clavulanate**	100	0	0
**Gentamicin**	71.4	0	28.6
**Amikacin**	71.4	14.3	14.3
**Meropenem (*n* = 6)**	66.7	0	33.3
***P*. *aeruginosa* (N = 2)**			
**Antimicrobial agent**	**Resistant (%)**	**Intermediate (%)**	**Sensitive (%)**
**Penicillin G (*n* = 1)**	100	0	0
**Piperacillin / tazobactam**	0	0	100
**Levofloxacin**	0	0	100
**Ciprofloxacin**	0	0	100
**Chloramphenicol**	100	0	0
**Gentamicin**	0	0	100
**Amikacin**	0	0	100
**Meropenem**	50	0	50
**Azetronam**	0	0	100
***N*. *meningitidis* (N = 2)**			
**Antimicrobial agent**	**Resistant (%)**	**Intermediate (%)**	**Sensitive (%)**
**Penicillin G**	100	0	0
**Ampicillin**	100	0	0
**Levofloxacin**	0	0	100
**Chloramphenicol**	0	0	100
**Cefotaxime**	100	0	0
**Ceftriaxone**	100	0	0
**Ciprofloxacin**	50	0	50
**Amikacin**	50	0	50
**Meropenem**	50	0	50

N, total number of isolates, *n*, number of tested isolates, R, resistant, I, intermediate, S, sensitive, %, percentage

### Identification of the multidrug-resistant (MDR) isolates

Out of the 71 isolates, 26 isolates (36.6%) were MDR isolates. Of these, 18 isolates (69.2%) were Gram-negative and 8 isolates (30.8%) were Gram-positive. Based on yellowish growth on mannitol salt agar and results of coagulase test of Gram positive isolate, 4 isolates were identified as *S*. *aureus* (50%), 3 isolates were coagulase negative *Staphylococci* (37.5%) and one isolate was *Enterococcus* (12.5%). *S*. *aureus* isolates (n = 6) were screened for methicillin resistance using cefoxitin disc diffusion method. The results showed that the six *S*. *aureus* isolates were methicillin sensitive *S*. *auerus*. (identified as”MSSA”). According to the microscopical, cultural and biochemical characteristics of the 18 MDR Gram-negative isolates, 7 (38.9%), 4 (22.2%), 2 (11.1%), 1 (5.5%), 1 (5.5%), 1 (5.5%), and 1 (5.5%) isolates were identified as *Acinetobacter spp*, *K*. *pneumoniae*, *E*. *coli*, *Citrobacter spp*, *P*. *aeruginosa*, *N*. *meningitidis* and *Proteus spp*, respectively.

### Antibiogram analysis of the MDR isolates

**Fig 3:**
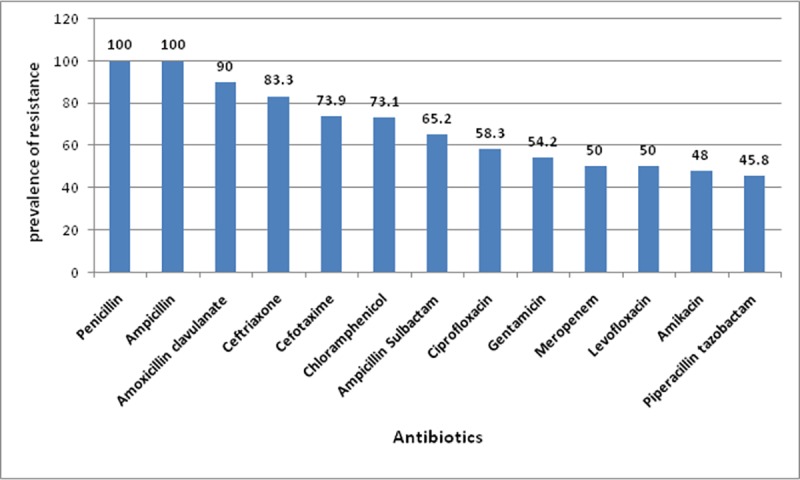
Prevalence of the antimicrobial resistance of the 26 tested MDR isolates to different antimicrobial agents. Prevalence was expressed as percent of resistant isolates relative to total tested isolates for each antimicrobial agent (n, 26).

As shown in Fig 3, all selected MDR isolates (n = 26) showed resistance to penicillin and ampicillin, their relative prevalence of resistance to other tested agents is shown in Tables 5 and 6.

**Table 5:** Antibiogram analysis of the MDR Gram negative isolates.

Nr	isolate	Antibiotics														
		AS	PT	Ci	CH	Pe	CT	Cx	AC	Ak	Le	No	Am	Ge	Az	Me
S 656	*K*. *pneumonia*e	R	R	R	R	R	R	R	R	R	R	R	R	R	R	R
S 988	*K*. *pneumonia*e	R	R	I	R	R	R	R	R	R	R		R	S	R	R
S 414	*K*. *pneumonia*e	S	S	S	R	R	R	R	R	S	S	S	R	S		S
S 907	*K*. *pneumonia*e	R	R	R	R	R	R	R	R	R	R		R	R		R
S 945	*Acinetobacter*	R	R	R	R	R	R	R	R	R	R	R	R	R		R
S 368	*Acinetobacter*	R	R	R	R	R	R	R	R	R	R	R	R	R		R
S 888	*Acinetobacter*	R	R	R	R	R	R	R	R	R	R	R	R	R		R
S 577	*Acinetobacter*	R	R	R	R	R	R	R	R	R	R	R	R	R		R
S 39	*Acinetobacter*	R	S	S	R	R	S	I		S	S	S	R	S		S
S 306	*Acinetobacter*	R	S	R	R	R	R	R	R	R	S	S	R	R		S
S 130	*Acinetobacter*	R	R	R	R	R	R	R	R	I			R	S		
S 414	*Citrobacter*	I	S	S	S	R	S	R	R	S	S	S	R	S	R	S
S 85	*Citrobacter*	S	S	S	S	R	S	S	S	S	S	S	R	S	R	S
S 85	*proteus*	R	S		R	R	R	R	R	S	I		R	I		S
S 220	*N*. *meningitidis*			R	S	R	R	R		R						R
S 282	*E*.*coli*	S	S	S	R	R	S	S	S	S	S	S	R	S		S
S 215	*E*.*coli*	R	S	S	S	R	R	R	R	R	I	S	R	I		S
S 184	*P*.*aeruginosa*		S	S	R	R				S	S	S	R	S	S	R

Abbreviations: AS, ampicillin-sulbactam; PT, Piperacillin-tazobactam; Ci, Ciprofloxacin; CH, Chloramphenicol; Pe, penicillin; CT, Cefotaxime; Cx, Ceftriaxone; AC, Amoxicillin -clavulanate; Ak, Amikacin; Le, Levofloxacin; No, Norfloxacin; Am, Ampicillin; Ge, Gentamicin, Az, Aztreonam; Me, Meropenem

**Table 6:** Antibiogram analysis of the MDR Gram positive isolates.

Nr	Isolate	Antibiotics													
		AS	PT	Ci	CH	Pe	CT	Va	Cx	AC	Ak	Le	Am	Ge	Me
S232	*S*. *aureus*	R	S	R	S	R		S	R	R	S	R	R	R	S
S353	*S*. *aureus*	I	S	S	R	R	S	R	R		S	S	R	S	S
S345	*S*. *aureus*	S	S	S	S	R	S	S	S	R	S	S	R	R	S
S516	*S*. *aureus*	S	S		R	R	R	S	R		S	S	R	R	S
S579	*S*. *coagulase-ve*	R	R	R	R	R	R	S	R	R	S	R	R	R	R
S836	*S*.*coagulase-ve*	S	R	R	R	R	R	R	R	R	R	R	R	R	R
S484	*S*. *coagulase-ve*	R	R	R	R	R	R	R	R	R	R	R	R	R	R
S110	*Enterococcus*			R	S	R		R				R	R		

Abbreviations: AS, ampicillin-sulbactam; PT, Piperacillin-tazobactam; Ci, Ciprofloxacin; CH, Chloramphenicol; Pe, penicillin; CT, Cefotaxime; Va, vancomycin; Cx, Ceftriaxone; AC, Amoxicillin-clavulanate; Ak, Amikacin; Le, Levofloxacin; Am, Ampicillin; Ge, Gentamicin, Me, Meropenem

### MIC results of some selected MDR isolates against some tested antimicrobial agents

The MIC results of some selected Gram negative and Gram positive MDR isolates are shown in Tables 7 & 8. The MDR *K*. *pneumoniae* and *Acinetobacter spp* isolates showed MIC values against ceftriaxone several fold shigher than 2μg/ml, therefore, they were considered as potential ESBLs producers according to the CLSI guidelines. For the MDR Gram negative isolates, the lowest resistance was observed to imipenem.

**Table 7:** MIC results for some selected MDR Gram-negative isolates (n = 15) against some selected antimicrobial agents.

Isolate	Bacterial species	MIC value (μg/ml)against:							
		AS	Cz	Cx	Am	Ge	Im	Cp	Le
S39	*Acinetobacter spp*	**32\16**	16	32	2	1	2	8	2
		**R**	I	I	S	S	S	S	S
S945	*Acinetobacter spp*	**32\16**	16	**128**	**64**	**16**	1	**32**	**16**
		**R**	I	**R**	**R**	**R**	S	**R**	**R**
S577	*Acinetobacter spp*	**32\16**	**32**	**64**	**512**	**128**	1	**32**	**64**
		**R**	**R**	**R**	**R**	**R**	S	**R**	**R**
S368	*Acinetobacter spp*	16\8	16	**64**	**512**	**128**	4	16	**64**
		I	I	**R**	**R**	**R**	S	I	**R**
S888	*Acinetobacter spp*	**64\32**	**32**	**256**	**64**	**64**	8	**32**	**64**
		**R**	**R**	**R**	**R**	**R**	I	**R**	**R**
S306	*Acinetobacter spp*	16\8	**32**	**64**	**64**	-	1	8	2
		I	**R**	**R**	**R**	-	S	S	S
S656	*K*. *pneumoniae*	**256\128**	**32**	**>64**	**64**	**16**	**16**	**32**	**16**
		**R**	**R**	**R**	**R**	**R**	**R**	**R**	**R**
S988	*K*. *pneumoniae*	**256\128**	**128**	**64**	**512**	2	2	**256**	**16**
		**R**	**R**	**R**	**R**	S	I	**R**	**R**
S907	*K*. *pneumoniae*	**>256\128**	**128**	**>64**	**64**	-	**8**	**>256**	**8**
		**R**	**R**	**R**	**R**	-	**R**	**R**	**R**
S282	*E*. *coli*	8\4	**32**	**16**	16	4	0.5	8	4
		S	**R**	**R**	S	S	S	S	I
S215	*E*. *coli*	16\8	**256**	**>64**	**512**	**32**	0.5	**256**	**8**
		I	**R**	**R**	**R**	**R**	S	**R**	**R**
S85	*Citrobacter spp*	8\4	**16**	**16**	16	8	0.5	0.5	2
		S	**R**	**R**	S	I	S	S	S
S414	*Citrobacter spp*	16\8	**64**	**64**	16	4	0.5	16	4
		I	**R**	**R**	S	S	S	I	I
S85	*Proteus spp*	**64\32**	**16**	**64**	32	**16**	0.5	1	**8**
		**R**	**R**	**R**	I	**R**	S	S	**R**
S184	*P*. *aeruginosa*		4	-	4	0.5	8	4	1
			S	-	S	S	I	S	S

Abbreviations: AS, ampicillin-sulbactam; Cz, Ceftazidime; Cx, Ceftriaxone; Ak, Amikacin; Ge, Gentamicin, Im, Imipenem; Cp, Cefepime; Le, levofloxacin

**Table 8:** MIC results for some selected MDR Gram-positive isolates (n = 5) against some selected antimicrobial agents.

Nr	Isolate	MIC value (μg/ml) against							
		AS	Do	Cx	Ak	Va	Im	Cp	Le
S 516	*S*. *aureus*	8\4	**64**	**64**	16	8	2	8	2
		S	**R**	**R**	S	I	S	S	I
S 353	*S*. *aureus*	16\8	**16**	**64**	16	**64**	1	16	**4**
		I	**R**	**R**	S	**R**	S	I	**R**
S 345	*S*. *aureus*	2	**16**	2	4	2	0.25	8	1
		S	**R**	S	S	S	S	S	S
S 484	*S*. *coagulase-ve*	**32\16**	**32**	**128**	**512**	**32**	-	**64**	**>128**
		**R**	**R**	**R**	**R**	**R**	-	**R**	**R**
S 836	*S*. *coagulase-ve*	**64\32**	**16**	**512**	**>512**	**256**	1	16	**32**
		**R**	**R**	**R**	**R**	**R**	S	I	**R**

Abbreviations: AS, ampicillin-sulbactam; Do, doxycycline;; Cx, Ceftriaxone; Ak, Amikacin; Va, vancomycin; Im, Imipenem; Cp, Cefepime; Le, Levofloxacin.

### MIC results of certain antibiotic combinations for some selected MDR Gram-negative isolates (n = 10)

Ten MDR Gram-negative isolates were selected to be tested against four different antibiotic combinations, and fractional inhibitory concentration (FIC) values were calculated for each isolate. It was found that ampicillin/sulbactam+cefepime combination showed synergism against 8 tested isolates (80%) and additive effect against 2 isolates (20%). Results of the four tested antibiotic combinations including: ampicillin/sulbactam+cefepime; ampicillin/sulbactam+amikacin; ampicillin/sulbactam+ levofloxacin; amikacin + levofloxacin are shown in Tables 9, 10, 11 and 12, respectively.

**Table 9:** FIC values of Ampicillin/sulbactam plus Cefepime combination.

Isolate code	Isolate	Ampicillin/sulbactam		Cefepime		Combination	FIC value	Interpretation
S85	*Proteus spp*	64/32	R	1	S	0.5/0.5	0.5	Synergism
S215	*E*. *coli*	16/8	I	256	R	1/1	0.066	Synergism
S414	*Citrobacter spp*	16/8	I	16	I	8/8	1	Additive
S656	*K*. *pneumoniae*	256/128	R	32	R	16/16	0.562	Additive
S907	*K*. *pneumoniae*	>256/128	R	>256	R	32/32	0.25	Synergism
S988	*K*.*pneumoniae*	256/128	R	256	R	16/16	0.125	Synergism
S888	*Acinetobacter spp*	64/32	R	32	R	8/8	0.375	Synergism
S945	*Acinetobacter spp*	32/16	R	32	R	4/4	0.25	Synergism
S368	*Acinetobacter spp*	16/8	I	16	I	4/4	0.5	Synergism
S577	*Acinetobacter spp*	32/16	R	32	R	8/8	0.5	Synergism

**Table 10:** FIC values of Ampicillin/sulbactam plus amikacin combination.

Isolate code	Isolate	Ampicillin/ sulbactam		Amikacin		Combination	FIC value	Interpretation
S85	*Proteus spp*	64/32	R	32	I	16/32	1.25	Indifference
S215	*E*. *coli*	16/8	I	512	R	64/128	4.25	Antagonism
S414	*Citrobacter spp*	16/8	I	16	S	2/4	0.375	Synergism
S656	*K*. *pneumoniae*	256/128	R	64	R	128/256	4.5	Antagonism
S907	*K*. *pneumoniae*	>256/128	R	64	R	128/256	4.5	Antagonism
S988	*K*. *pneumoniae*	256/128	R	512	R	64/128	0.5	Synergism
S888	*Acinetobacter spp*	64/32	R	64	R	128/256	6	Antagonism
S945	*Acinetobacter spp*	32/16	R	64	R	32/64	2	Indifference
S368	*Acinetobacter spp*	16/8	I	512	R	128/256	8.5	Antagonism
S577	*Acinetobacter spp*	32/16	R	512	R	128/256	4.5	Antagonism

**Table 11:** FIC values of Ampicillin/sulbactam plus Levofloxacin combination.

code	Isolate	Ampicillin/ sulbactam		levofloxacin		Combination	FIC value	Interpretation
S85	*Proteus spp*	64/32	R	8	R	64/32	5	Antagonism
S215	*E*. *coli*	16/8	I	8	R	64/32	8	Antagonism
S414	*Citrobacter spp*	16/8	I	4	I	8/4	1.5	Indifference
S656	*K*.*pneumoniae*	256/128	R	16	R	128/64	4.5	Antagonism
S907	*K*. *pneumoniae*	>256/128	R	8	R	32/16	2.125	Indifference
S988	*K*. *pneumoniae*	256/128	R	16	R	32/16	1.125	Indifference
S888	*Acinetobacter spp*	64/32	R	64	R	128/64	3	Indifference
S945	*Acinetobacter spp*	32/16	I	16	R	64/32	4	Indifference
S368	*Acinetobacter spp*	16/8	I	64	R	64/32	4.5	Antagonism
S577	*Acinetobacter spp*	32/16	R	64	R	128/64	5	Antagonism

**Table 12:** FIC values of Amikacin plus Levofloxacin combination.

Isolate code	Isolate	Amikacin		Levofloxacin		Combination	FIC value	Interpretation
S85	*Proteus spp*	32	I	8	R	128/32	8	Antagonism
S215	*E*. *coli*	512	R	8	R	64/16	2.125	Indifference
S414	*Citrobacter spp*	16	S	4	I	32/8	4	Indifference
S656	*K*. *pneumoniae*	64	R	16	R	25/64	8	Antagonism
S907	*K*. *pneumoniae*	64	R	8	R	64/16	3	Indifference
S988	*K*.*pneumoniae*	512	R	16	R	64/16	1.125	Indifference
S888	*Acinetobacter spp*	64	R	64	R	256/64	5	Antagonism
S945	*Acinetobacter spp*	64	R	16	R	64/16	2	Indifference
S368	*Acinetobacter spp*	512	R	64	R	256/64	1.5	Indifference
S577	*Acinetobacter spp*	512	R	64	R	256/64	1.5	Indifference

**R:** resistance, **I:** intermediate, **S:** sensitive. **FIC:** fractional inhibitory conc. **Synergism**≤0.5, **Additive**≤1,**Indifference** >1and≤4.0, **Antagonism**>4.

### MIC results of some selected antibiotic combination for two MDR Gram-positive isolates:

MDR Gram positive bacteria, 2 isolates were tested against five different antibiotic combinations and the FIC values were calculated for each combination. Synergism was observed with doxycycline+levofloxacin combination. Table 13 shows the results of the antibiotic combinations used.

**Table 13:** FIC values calculated for 5 different antibiotic combinations for MDR Gram-positive isolates.

**For Ampicillin/sulbactam plus Cefepime combination**						
**Isolate code**	**Bacterial species**	**Ampicillin/ sulbactam**	**Cefepime**	**Combination**	**FIC value**	**Interpretation**
S345	*S*.*aureus*	2/1	8	4/4	2.5	Indifference
S836	*S*. *coagulase*-ve	64/32	16	8/8	0.62	Additive
**For Vancomycin plus Levofloxacin combination**						
		**Vancomycin**	**Levofloxacin**			
S345	*S*.*aureus*	2/1	1	4/2	4	Indifference
S836	*S*. *coagulase*-ve	256	32	128/64	2.5	Indifference
**For Doxycycline plus Levofloxacin combination**						
		**Doxycycline**	**Levofloxacin**			
S345	*S*.*aureus*	16	1	0.25/0.25	0.26	Synergism
S836	*S*. *coagulase*-ve	16	32	4/4	0.37	Synergism
**For Ampicillin/sulbactam plus Vancomycin combination**						
		**Ampicillin/ sulbactam**	**Vancomycin**			
S345	*S*.*aureus*	2/1	2	4/4	4	Indifference
S836	*S*. *coagulase*-ve	64/32	256	64/64	1.25	Indifference
**For Doxycycline plus Amikacin combination**						
		**Doxycycline**	**Amikacin**			
S345	*S*.*aureus*	16	4	2/8	2.125	Indifference
S836	*S*. *coagulase*-ve	16	512	4/16	0.28	Synergism

*S*. *aureus*, *Staphylococcus aureus*; *S*. *coagulase-ve*,coagulase negative *Staphylococcus;* R: resistant, I: intermediate, S:sensitive. FIC: fractional inhibitory conc. Synergism≤0.5, Additive≤1, Indifference> 1 and ≤4.0, Antagonism>4.

### Detection of antibiotic resistance genes using PCR

**Fig 4:**
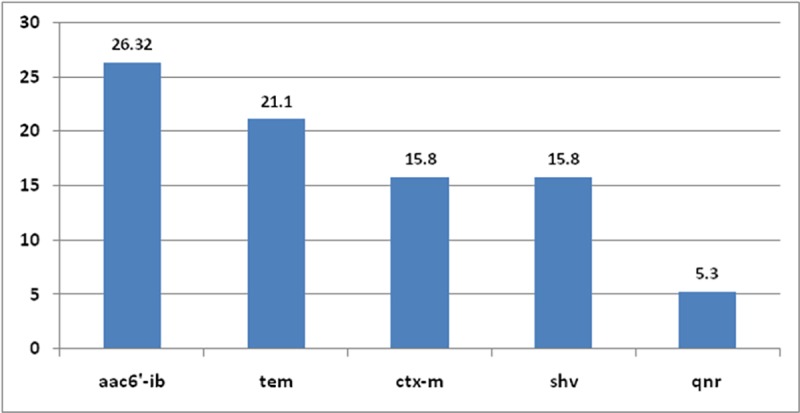
Prevalence of some selected antibiotic resistance genes among MDR bacterial pathogens.

Plasmid bands were not detected in any of tested 19 MDR isolates, however, when subjected to PCR gene detection, only 9 isolates gave positive results (47.36%).The PCR products of *tem*, *shv*, *ctx-m* obtained from chromosomal DNA of *K*. *pneumoniae* isolate S907 and the PCR product of *aac(6')* obtained from using chromosomal DNA of *Acinetobacter spp*, isolate S888 were analyzed, annotated and submitted into the GenBank database under accession numbers KX214665,KX214664,KX214663, KX214662, respectively. Prevalence of resistance genes investigated were: *tem* (21.1%), *shv* (15.8%), *ctx-m* (15.8%) coding TEM-, SHV, CTX-M extended spectrum beta-lactmases (ESBLs), respectively; *aac*(6')-*Ib* (26.32%) coding for aminoglycoside 6’-N-acetyltransferase type Ib ciprofloxacin resistant variant; and *qnr*A (5.3%) gene coding for quinolone resistance (Fig 4). The genotypic characteristics of the MDR resistant isolates were also analyzed (Table 14).

**Table 14:** Genotypic detection of resistant gene using chromosomal DNA.

Specimen NO	Isolate	*ctx-m*	*shv*	*tem*	*aac*(6)-ib\ibcr	*qnr*
S 184	*P*. *aeruginosa*	**-**	**-**	**-**	**-**	**nd**
S 656	*K*. *pneumoniae*	**-**	**+**	**-**	**-**	**-**
S 907	*K*. *pneumoniae*	**+**	**+**	**+**	**-**	**-**
S 988	*K*. *pneumoniae*	**-**	**-**	**-**	**-**	**-**
S 414	*K*. *pneumoniae*	**-**	**-**	**-**	**nd**	**nd**
S 888	*Acinetobacter spp*	**-**	**-**	**+**	**+**	**+**
S 577	*Acinetobacter spp*	**-**	**+**	**-**	**+**	**-**
S 368	*Acinetobacter spp*	**-**	**-**	**+**	**-**	**-**
S 945	*Acinetobacter spp*	**+**	**-**	**+**	**+**	**-**
S 306	*Acinetobacter spp*	**-**	**-**	**-**	**-**	**-**
S130	*Acinetobacter spp*	**-**	**-**	**-**	**-**	**nd**
S 414	*Citrobacter spp*	**-**	**-**	**-**	**nd**	**nd**
S 282	*E*. *coli*	**-**	**-**	**-**	**nd**	**nd**
S 215	*E*. *coli*	**+**	**-**	**-**	**-**	**-**
S 85	*Proteus spp*	**-**	**-**	**-**	**-**	**-**
S 220	*N*. *meningitidis*	**-**	**-**	**-**	**+**	**-**
S836	*S*. *coagulase-ve*	**-**	**-**	**-**	**+**	**-**
S 484	*S*. *coagulase-ve*	**-**	**-**	**-**	**-**	**-**
S 579	*S*. *coagulase-ve*	**-**	**-**	**-**	**nd**	**-**

(-), absent, (+), present, nd, not detected.

## Discussion

Bacterial meningitis continues to be a potentially life threatening disease with substantial morbidity and mortality throughout the world. It represents even more significant problem in many other regions of the world, especially in developing countries [1,25]. The uncontrolled use of antibiotics and their overuse lead to rapid and extensive spread of antimicrobial resistance [26]. Antimicrobial resistance (AMR) limits effective treatment and prevention of the increasing range of infections caused by bacteria, which represents critical threat to global public health, as new antimicrobial resistance mechanisms continue to emerge and spread globally [26]. It is obvious that bacterial pathogens will continue to develop resistance against currently available antibacterial agent either through developing newly genetic mutations or exchange of genetic information.

In the current study, a total of 71 clinical bacterial isolates were recovered from CSF specimens collected through two years period from patients with bacterial meningitis. These clinical specimens were CSF. Using Gram-stain, 48 isolates (67.6%) were found to be Gram-positive and 23 isolates (32.4%) were found to be Gram-negative, From 48 Gram positive isolates the majority were *S*. *pneumoniae* constituting 31 isolates (64.6%,n = 48) indicating that Gram positive bacteria mainly *pneumococcal* meningitis responsible for highest contribution to bacterial meningitis in Egypt. Comparing our results with a study conducted also in Egypt it was found that *S*. *pneumoniae* was recently described as the leading cause of bacterial meningitis [27–29], reflecting a change in disease epidemiology since *N*. *meningitides* was for a long time the main causative pathogen of bacterial meningitis [30–34].Results obtained in this study where Gram positive bacterial were of the highest prevalence, particularly *Staphylococci* and *Streptococcus(S*.*) pneumoniae* were in accordance to those obtained from previous studies conducted in USA hospital laboratories from 2000–2002 [35]as well as from a teaching hospital in Ghana[36].

In another prospective, multicenter, observational study of 156 consecutive adults hospitalized for pneumococcal meningitis, *S*.*pneumoniae* was found to be the most common bacterium isolated from adults with community- acquired meningitis, [37]. The antimicrobial susceptibility testing of the Gram-positive isolates collected in this study (n = 48) showed that the lowest resistance was observed to meropenem, ampicillin/sulbactam and piperacillin/tazobactam only 3 isolates (8.57%, 8.57%, 9.1%) were resistant to each. Contrary to that, the highest resistance was observed with penicillin where 21 isolates (44.7%) showed resistance. The antimicrobial susceptibility testing of the 23 Gram-negative isolates collected in this study showed that the lowest resistance was observed to levofloxacin and gentamicin. Thus both bacterial categories recorded low resistance pattern to meropenem, levofloxacin and piperacillin/tazobactam. The highest resistance was observed to penicillin and ampicillin where 41 isolates (61.2%) were resistant to penicillin and 32 isolates (51.6%) were resistant to ampicillin. Several studies reported that penicillin resistant *S*. *pneumonia* showing an increase in resistance pattern over time [28, 30, 38–40]. However, in the current study only 7 isolates out of 31 tested isolates of *S*. *pneumoniae* were resistant to penicillin.

In another study conducted in Lebanon, among 44 tested *S*. *pneumoniae* isolates using E-tests 35 isolates were resistant to penicillin [41]. Among the 71 collected isolates, 26 isolates (36.6%) were found to be resistant to three or more antimicrobial classes, 18 of these isolates were Gram-negative (69.2%), while 8 isolates were Gram-positive (30.8%). For MDR Gram negative isolates the highest resistance was observed to penicillin and ampicillin, the lowest resistance was observed to levofloxacin, gentamicin and piperacillin/tazobactam. For MDR Gram positive isolates the highest resistance was observed to penicillin and ampicillin and lowest resistance was observed to amikacin, piperacillin/tazobactam, and meropenem. It can be concluded that MDR organisms will evolve on a continuous basis, compromising antimicrobial efficacy and will represent a treatment challenge for microbial infections.

Multidrug-resistant *Acinetobacter spp* was the most prevalent Gram-negative MDR pathogen (n = 7/26; 27%). All tested isolates showed resistance to ampicillin/sulbactam, chloramphenicol, penicillin and ampicillin. However, resistance was absent to imipenem where none of the isolates (0%) were resistant. Comparing our results with a study conducted in India stating that since 1975, increasing antimicrobial resistance against *Acinetobacter* started to appear in almost all groups of antibiotics including the first and second generation cephalosporins. Initially *Acinetobacter* isolates retained partial susceptibility against the third and fourth generation cephalosporins, fluoroquinolones, aminoglycosides, and carbapenems, with almost 100% isolates retaining susceptibility towards imipenem [42–44]. By the late 1990s, carbapenems were the only valuable agents remaining that could treat many severe *Acinetobacter* infections. However, due to clonal spread of carbapenem resistance *Acinetobacter baumannii* strains, the therapeutic options are decreasing [45–47]. This resistance has been found to be attributed to various mechanisms [48].

Along with our study, a study conducted in USA revealed that the response of *Acinetobacter spp* isolates to the combination of cefepime and ampicillin/sulbactam showed that no antagonistic interactions were recorded. Nine isolates (26.5%) showed synergism (FIC, ≤0.5), 21 isolates (61.8%) showed partial synergism. While four isolates (11.8%) showed an additive effect. Cefepime MIC values were ≤8 mg/L to 85.3% of strains, while ampicillin/sulbactam MIC values were ≤16/8 mg/L (intermediate) for all tested isolates when these antibiotics were tested in combination [49]. In a study conducted in China to investigate the mechanism of drug resistance of carbapenems-resistant *Acinetobacter baumannii* (CRAB) in burn patients, the antimicrobial activity of drugs combination against these bacteria in *vitro* showed that, the synergic, additive, indifferent, and antagonistic effects were respectively observed in 40, 33, 6, and 15 strains applied with combination of amikacin and ampicillin/sulbactam [50]. While in the current study out of 4 tested *Acinetobacter* isolates none showed synergism, 3 isolates showed antagonism while 1 isolate showed indifference. In another study conducted in USA two clinical strains of carbapenemase (KPC)–producing *K*. *Pneumoniae* were investigated. Various combinations including amikacin, doripenem, levofloxacin, and rifampin were quantitatively assessed, time-kill studies were conducted using clinically relevant concentrations and it was found that, amikacin plus levofloxacin was found to have antagonistic effect, [51]. While in the current study out of 3 tested MDR *K*. *pneumoniae*, one isolate showed antagonism while the two other isolates showed indifference. This reveals the significance of avoiding empirical selection of antimicrobial combinations, particularly for infections involving MDR organisms in which high mortality may already be likely [52]

The detection of genetic determinants associated with MDR resistance isolates, plasmid bands were absent in 19 tested MDR isolates. While extracted DNA of 19 isolates (16 Gram negative and 3 Gram positive MDR isolates) using PrepMan Ultra kit in an attempt to detect resistance genes from bacterial chromosome. Out of MDR 19 isolates subjected to PCR gene detection only 9 isolates gave positive results (47.36%), other isolates showed negative results despite their positive resistance phenotype. In a study conducted also in china, MDR *K*. *pneumoniae* strains isolated from patients in intensive care units (ICUs) demonstrated β-lactamase genes, such as *blaSHV* (22/38), *blaTEM* (10/38) and *blaCTX-M* (7/38) [53]. While In the present study out of the four tested MDR *K*. *pneumoniae*, resistance genes were only detected in two isolates, both isolates harbored *shv* gene, while *ctx-m* and *tem* genes were detected in only one of them.

A population-based laboratory surveillance study of ESBL-producing *E*. *coli* infections conducted in Canada reported that 70% of the ESBL in *E*. *coli* isolated were of the CTX-M-type. [54]. A study by Jorgensen et al. conducted in 2007, indicated that CTX-M-type ESBLs had emerged in *E*. *coli* and other species of *Enterobacteriaceae* in USA [55]. Moreover, recent studies from Philadelphia, Chicago, and Pittsburgh revealed a high prevalence of CTX-M-producing *E*. *coli* ST131 in adult patients [56–58]. However, despite extensive studies of ESBL-producing microorganisms there is a lack of information regarding development and spread of ESBL-mediated resistance, specifically CTX-M, in Gram-negative infections [59]. In the present study from two MDR *E*. *coli* only one isolate expressed *ctx*-*m* gene. CTX gene was also found in one *Acinetobacter* and *K*.*pneumoniae* isolates. Quinolone resistance is mainly mediated through chromosomal mutations in bacterial topoisomerase genes or genes that regulate the expression of efflux pumps [60,61]. In a study conducted in the USA, 313 *Enterobacteriaceae* isolates including *E*. *coli*, *K*. *pneumoniae* and *Enterobacter* with ciprofloxacin MIC of ≥0.25 μg/ml and reduced susceptibility to ceftazidime, were tested to detect presence of *aac*(6′)-Ib and *qnr* genes. The results indicated the presence of *aac*(6′)-Ib in 50.5% of isolates, and of these isolates, 28% carried the *cr* variant associated with low-level ciprofloxacin resistance. Moreover, *aac*(6′)-Ib-cr gene proved to have geographic widespread, stability over time, most prevalent in *E*. *coli* and not associated with presence of *qnr* genes [62]. However in the current study *aac*(6′)-Ib was detected in 4 MDR Gram negative isolates and one coagulase negative *Staphylococcus* isolate, but it was absent in tested MDR *K*. *pneumoniae* and *E*. *coli* isolates. Plasmid-borne quinolone resistance *qnr* genes have been found in clinical isolates of *Enterobacteriaceae* [63]. In the current study *qnr* gene was detected in only one MDR *Acinetobacter* isolate, as *qnr* genes is most predominant in acquired plasmid rather than bacterial chromosome.

## Conclusion

Piperacillin/tazobactam, levofloxacin, meropenem and ampicillin/sulbactam had a favorable sensitivity pattern among Gram-positive and Gram-negative pathogens; thus they are recommended for treatment of bacterial meningitis. MIC results showed that lowest resistance for MDR gram negative isolates was to imipenem, however its epiliptogenic side effect limits its use in meningitis especially in pediatrics. It was found that FIC values of ampicillin/sulbactam plus cefepime combination gave either synergism or additive effect against MDR Gram negative isolates, thus it is recommended to be used as a treatment option. While MDR Gram positive isolates showed synergism with doxycycline plus levofloxacin combination. Genotypic analysis detected that the antibiotic resistance of bacteria causing bacterial meningitis is mainly chromosomal mediated, several resistant genes were detected including ESBLs (*tem*, *ctx-m*, *shv*) accounting for resistance to β-lactam antibiotics, also *aac*(6')-Ib resistance gene accounting for resistance to aminoglycoside (amikacin). It is recommended to monitor drug-resistant isolates and consider rational use of antimicrobials agents in order to limit the spread and prevalence of the underlying resistance mechanisms.

## Abbreviations

FIC: fractional inhibitory concentration
*tem*, *shv*, *ctx*-*m*: genes coded for TEM-, SHV, CTX-M extended spectrum beta-lactmases, respectively
ESBLs: extended-spectrum beta-lactmases
aac(6')-Ib: gene coded for aminoglycoside 6’-N-acetyltransferase type Ib ciprofloxacin resistant variant
*qnr*A: gene coded for quinolone resistance
CSF: cerebrospinal fluid
MICs: minimum inhibitory concentrations
Ta: calculated annealing temperatures
AMR: Antimicrobial resistance
PCR: polymerase chain reaction
